# Effect of recombinant Hepatitis B virus on human glomerular mesangial cell apoptosis

**DOI:** 10.1080/13102818.2014.948278

**Published:** 2014-10-22

**Authors:** Yiguo Wang, Changhong Liu, Sen Hong, Pengju Zhang, Qian Liu

**Affiliations:** ^a^ Department of Gastroenterology, Qianfoshan Hospital affiliated to Shandong University, Jinan, Shandong, P. R. China; ^b^ Department of Internal Medicine, Shandong Academy of Medical Sciences, Jinan, Shandong, P. R. China; ^c^ Institute of Biochemistry and Molecular Biology, Medical School of Shandong University, Jinan, Shandong, P. R. China

**Keywords:** Hepatitis B virus, glomerular mesangial cell, apoptosis, Caspase-3

## Abstract

The aim of this study was to investigate the expression of recombinant Hepatitis B virus (HBV) in normal human glomerular mesangial cells (NHMC) and its effect on cell apoptosis. Cell transfection was conducted by the liposome method. The levels of HBsAg and HBeAg in the culture supernatant were detected by electrochemiluminescence. Morphological changes were observed by light and fluorescence microscopy. Cell proliferation was analysed by the methyl thiazole tetrazolium (MTT) assay and cell apoptosis, by flow cytometry. The expression level of Bax and Bcl-2 mRNA was measured by semi-quantitative reverse-transcription polymerase chain reaction (RT-PCR). Caspase-3 activity was detected by a Caspase-3 activity detection kit. The results showed high expression levels of HBsAg and HBeAg in NHMC cells transfected with recombinant full-length C genotype HBV (PHY106-CHBV). Typical apoptotic morphology was observed at 48 h after PHY106-CHBV transfection. Cell proliferation was inhibited. The percentage of apoptotic cells and the expression level of Bax mRNA were significantly higher in the PHY106-CHBV group than those in the blank control group and the PHY106 group. There was no significant difference in the expression level of Bcl-2 mRNA among the three groups. Caspase-3 was significantly activated after PHY106-CHBV transfection. The results demonstrate that recombinant HBV can be expressed in NHMC and its expression induces NHMC apoptosis.

## Introduction

Hepatitis B virus associated glomerulonephritis (HBV-GN) is one of the common extrahepatic damages caused by HBV viral infection. The cause of HBV-GN is generally believed to be immune-mediated damage by immune complex deposition in renal tissues. However, recent studies suggest that a direct HBV infection in renal cells and tissues may be correlated with renal pathological alterations. For example, HBV DNA was detected in renal cells and tissues such as tubular epithelial cells, glomerular epithelial cells, glomerular mesangial cells and the glomerular basement membrane by *in situ* hybridization.[1,2] Immunoelectron microscopy detected virus-like particle structures distributed in the epithelial cells, basement membrane and mesangial area in the renal tissues of HBV-GN patients.[1–3] Based on these studies, an HBV direct pathogenesis theory is proposed, i.e. HBV infects and replicates *in situ* in renal tissues of HBV-GN patients, causing kidney damage. However, there has been no direct experimental evidence to support this theory.

Studies have shown that apoptosis may be involved in virally induced renal pathogenesis. For example, Deng et al. [4] incubated renal tubular epithelial cells (HK-2) with the serum of patients with chronic hepatitis B and found that the apoptosis rate was significantly higher in HBV serum groups than those in control groups. In addition, the apoptosis rate of HK-2 in the HBV DNA positive serum group was significantly higher than that in HBV DNA negative serum. In addition, the apoptosis rate of HK-2 cells in HBV DNA positive serum group was positively correlated with the level of HBV DNA. These data suggest a role of HBV in renal tubular cell apoptosis. Hong et al. [5] found that cells transfected with *mhbs* ^t167^ and/or *hbx* increased NF-κB nuclear translocation, phosphor-IκBα, κB-DNA-binding activity, κB-dependent transcription and apoptotic index compared to controls. These data provide evidence that HBV infection may also cause renal damage by inducing apoptotic cell loss.

In spite of our increased understanding of mechanisms potentially involved in HBV infection-induced apoptotic cell loss in HBV-GN, how apoptotic changes are observed during HBV expression in renal glomerular mesangial cells has been scarcely studied. In this study we investigated cell apoptosis in normal human glomerular mesangial cells (NHMC) by ectopically expressing a recombinant full-length C genotype HBV.

## Materials and methods

### Cell line and cell culture

The NHMC cell line was obtained from the Institute of Biochemistry and Molecular Biology, Medical School of Shandong University, Jinan. Cells were cultured in low glucose Dulbecco's Modified Eagle's medium (DMEM; Gibco, Invitrogen, Carlsbad, California, USA) supplemented with 10% fetal bovine serum (Zhejiang Tianhang Biotechnology Co., Ltd., Hangzhou, China) and kept in a humidified incubator (5% CO_2_) at 37 ºC.

### Transfection

Recombinant full-length C genotype HBV plasmid PHY106-CHBV was kindly provided by Prof. Chengjun from Beijing Ditan Hospital. Plasmid transfection was performed using Lipofectamine^TM^ 2000 (Invitrogen, Carlsbad, California, USA) following manufacturer's protocol. Briefly, one day before transfection, NHMC cells were seeded in 24-well plate at a concentration of 1 × 10^5^ per well. When cell confluence was over 70%, cells were transfected with a mixture of plasmid DNA, Lipofectamine^TM^ 2000 and optimized Minimal Essential Medium (Opti-MEM). Cells were divided into three groups. The blank control group was transfected without DNA. The negative control group (the PHY106 group) was transfected with PHY106 vector. The test group (the PHY106-CHBV group) was transfected with PHY106-CHBV plasmid. At 24, 36, 48 and 72 h after transfection, cells were processed and analysed as described for each experiment.

### Electrochemiluminescence (ECL)

Quantitative detection of HBsAg and HBeAg in the supernatant was performed using ECL by Clinical Laboratory of Qianfoshan Hospital affiliated to Shandong University, Jinan. The supernatant of NHMC cell culture was collected at 24, 48 and 72 h after transfection. The experiments were performed using a cobas e 601 automatic analyser (Roche, Basel, Switzerland), according to the procedure suggested by the manufacturer.

### Cell proliferation by MTT assay

The number of living cells was determined by the methyl thiazole tetrazolium (MTT) assay. Briefly, at 36 h after transfection, cells were collected and re-plated at the same density per well onto 96-well plates. Six duplicate wells and blank control wells with cell culture medium alone were set up for each group. MTT reagent (10 μL, Sigma, St. Louis, Missouri, USA) was added to each well and incubated for 4 h at 37 °C. After incubation, the culture supernatant was removed and 100 μL of dimethyl sulfoxide (DMSO; Sigma, St. Louis, Missouri, USA) was added to each well. After oscillating for about 10 min, the absorbance values of the wells, including the blanks, were measured at 570 nm with a reference wavelength of 650 nm on a Model 680 microplate reader (Bio-Rad, Hercules, California, USA).

### Morphological evaluation by Hoechest 33258 staining

For identification of cells with nuclear morphology of apoptosis, cells were stained with the DNA dye Hoechst 33258 (Sigma, St. Louis, Missouri, USA). At 48 h after transfection, cells were fixed on the 24-well plate for 10 min with 300 μL of 4% paraformaldehyde. After washing with phosphate-buffered saline (PBS) twice, cells were incubated in the dark with Hoechst 33258 for 5 min. After washing again with PBS twice, nuclear morphology was observed under a fluorescence microscope.

### Cell apoptosis analysis by Annexin V/PI Kit

Forty-eight hours after transfection, cultured cells were collected and washed once with cold PBS. According to the manufacture's protocol, cells were resuspended in 500 μL of 1× annexin-binding buffer at a concentration of 1 × 10^6^ cells and stained with 5 μL of Annexin V and 10 μL of propidium iodide (PI; BestBio, Jinan, China) in the dark for 5 min at 2–8 °C. After staining, cell apoptosis was analysed by flow cytometry (BD, San Jose, California, USA).

### Semi-quantitative reverse-transcription polymerase chain reaction (RT-PCR)

Forty-eight hours after transfection, cells were collected and total RNA was extracted using TRIzol (Invitrogen, Carlsbad, California, USA). For RT-PCR, RNA was reverse transcribed with M-MuLV (Fermentas, Vilnius, Lithuania) to form cDNA. The primer pairs for the PCR amplification of *Bax*, *Bcl-2* and *GAPDH* (glyceraldehyde 3-phosphate dehydrogenase) were synthesized by Sangon Biotech Co., Ltd (Shanghai, China) and their sequences are given in Table 1.

**Table 1.** Primer sequences used in reverse transcription-PCR.

Bax	F1: 5′AAGCTGAGCGAGTGTCTCAAG3′
	R1: 5′CAAAGTAGAAAGGGCGACAAC3′
Bcl-2	F1: 5′TGGGAGAACAGGGTACGATAAC3′
	R1: 5′GAACTCAAAGAAGGCCACAATC3′
GAPDH	F1: 5′GGGAAACTGTGGCGTGAT3′
	R1: 5′GAGTGGGTGTCGCTGTTGA3′

DNA polymerase rTaq (TaKaRa Biotechnology (Dalian) Co., Ltd., Dalian, China) was used to amplify *Bax*, *Bcl-2* and *GAPDH* from the template cDNA. GAPDH was used as an internal control. The PCR products of *Bax*, *Bcl-2* and *GAPDH* were analysed in a 1.5% agarose gel and scanned by a gel-imaging system (Shanghai Forte Co., Shanghai, China).

### Caspase-3 activity detection

The activity of Caspase-3 was determined using the Caspase-3 activity kit (Beyotime, Haimen, Jiangsu, China). Briefly, at 48 h after transfection, cells were collected and total protein was extracted. Total proteins were incubated with Caspase-3 substrate (Ac-DEVD-pNA) at 37 °C for 2 h. Samples were measured with a SpectraMax M2 reader at an absorbance of 405 nm.

### Statistical analysis

SPSS17.0 software was used for statistical analysis and the experimental data were expressed as 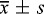. A *P*-value <0.05 was considered statistically significant.

## Results and discussion

### Recombinant full-length C genotype HBV is successfully cloned into PHY106 vector

**Figure 1.**
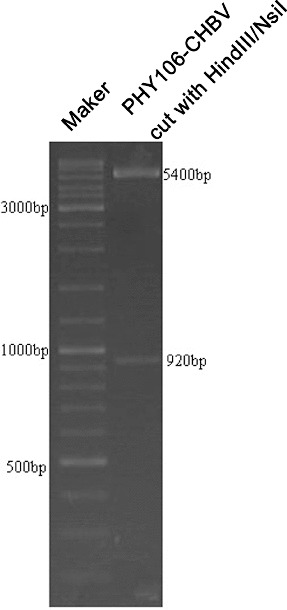
Endonuclease digestion of plasmid PHY106-CHBV. Two DNA fragments of 5400 bp and 920 bp were generated after endonuclease Hind III and Nsi I digestion.

**Figure 2.**
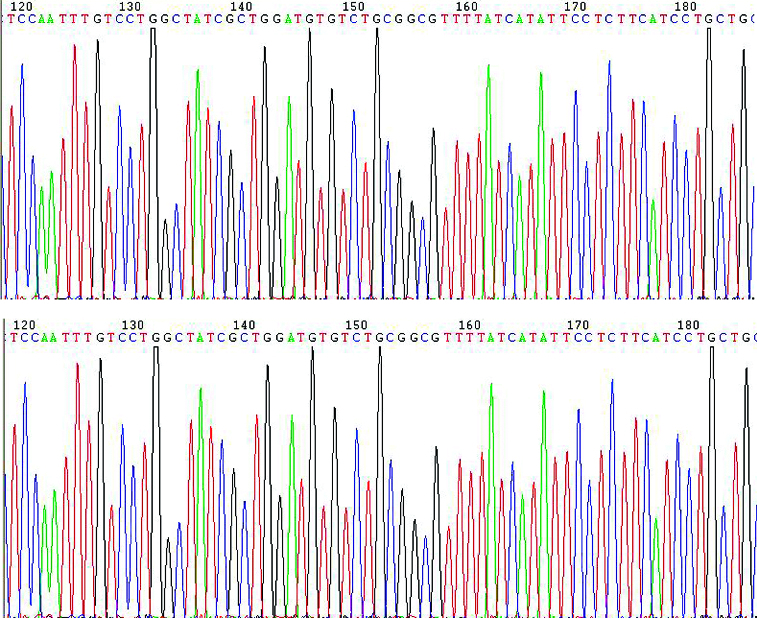
Gene sequencing map of the plasmid PHY106-CHBV. Part of the gene sequencing results are shown.

To confirm that PHY-l06 eukaryotic expression vector contains the genotype C HBV gene sequences, we used endonuclease digestion and gene sequencing. After digestion with endonuclease Hind III and Nsi I, two DNA fragments of 5400 and 920 bp were shown in the agarose gel (Figure 1). These two fragments were of the same length as the PHY106 vector and the genotype C HBV gene, suggesting that the C HBV gene was correctly cloned into the PHY106 vector. The gene sequencing results (Figure 2) further confirmed that the genotype C HBV gene sequences were correct.

### HBsAg and HBeAg are expressed in NHMC cell culture supernatant after PHY106-CHBV transfection

HBsAg and HBeAg are markers of HBV infection and expression. In order to study the expression of recombinant PHY106-CHBV in NHMC cells, we measured the levels of HBsAg and HBeAg in the culture supernatant. As shown in Table 2, at 24, 48 and 72 h after transfection, the expression of HBsAg and HBeAg in the blank control group and the PHY106 group was negative. At 24 h after transfection, HBsAg and HBeAg expression was also negative in the PHY106-CHBV group. However, at 48 and 72 h after transfection, the expression of HBsAg and HBeAg in the PHY106-CHBV group was positive. Statistically there was no significant difference between the two control groups (the blank control group and the PHY106 group) at all time points (*P* > 0.05). Also at 24 h after transfection, there was no significant difference among the three groups (*P* > 0.05). However, at 48 and 72 h after transfection, the levels of HBsAg and HBeAg in the PHY106-CHBV group were significantly higher than those in the control groups (*P* < 0.05). This result indicates that PHY106-CHBV can be expressed in NHMC cells.

**Table 2.** The expression levels of HBsAg and HBeAg in the culture supernatant analyzed by ECL at 24 h, 48 h and 72 h after transfection.

	The OD570 value of HBsAg			The OD570 value of HBeAg		
	24 h	48 h	72 h	24 h	48 h	72 h
The blank control group	0.10 ± 0.06	0.11 ± 0.02	0.13 ± 0.02	0.14 ± 0.02	0.18 ± 0.03	0.14 ± 0.02
The PHY106 group	0.11 ± 0.02	0.12 ± 0.01	0.11 ± 0.02	0.15 ± 0.03	0.13 ± 0.03	0.13 ± 0.03
The PHY106-CHBV group	0.16 ± 0.04	0.59 ± 0.08*	1.77 ± 0.91*	0.19 ± 0.02	1.08 ± 0.15*	1.64 ± 0.30*

Note: The quantitative biological reference interval for HBsAg and HBeAg was 0 to 0.2 ng/ mL and 0 to 0.5 PEI U/ mL, respectively.

* *P* < 0.05 (the PHY106-CHBV group vs the blank control group and the PHY106 group).

### PHY106-CHBV expression decreases the cell viability of NHMC

MTT assay is now widely accepted as a reliable way to measure cell proliferation and the reduction in cell viability. In the present study we analysed the effect of PHY106-CHBV expression on NHMC cell viability by MTT at 48 h after transfection. The OD570 values of the blank control group, the PHY106 group and the PHY106-CHBV group were 0.96 ± 0.03, 0.94 ± 0.03 and 0.52 ± 0.05, respectively. Statistically there was no significant difference between the two control groups (the blank control group and the PHY106 group) (*P* > 0.05). In contrast, the cell viability in the PHY106-CHBV group was significantly lower than that in the control groups (*P* < 0.05). The cell viability inhibition rate in the PHY106-CHBV group was 45.17% ± 4.203%. These data suggest that NHMC cell viability is reduced after PHY106-CHBV expression, leading to inhibited cell proliferation.

### PHY106-CHBV expression induces apoptotic cell morphology in NHMC cells

**Figure 3.**
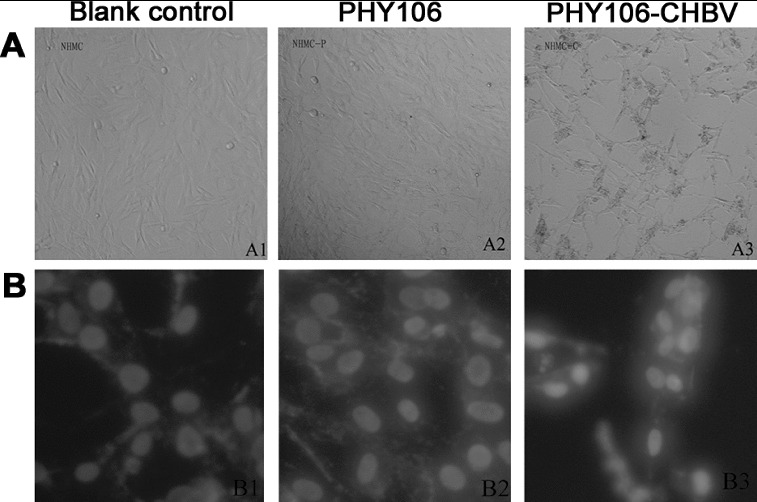
Effect of PHY106-CHBV expression on the morphology of NHMC cells 48 h after transfection. Representative photomicrographs taken under a light (100×) microscope (A) and a fluorescence (200×) microscope (B).

Apoptotic cell loss is one of the common reasons that cause cell viability reduction and cell proliferation inhibition. Thus, we examined whether PHY106-CHBV expression could induce apoptosis in NHMC cells. Since the criterion that best identifies apoptosis is the morphological one, we first checked cell morphology changes at 48 h after transfection. The normal morphology of NHMC cells is a stellate kite-like shape with oval nucleus and varying lengths of cytoplasm protrusions. As shown in Figure 3(A) (A1 and A2), the two control groups (the blank control group and the PHY106 group) showed normal NHMC cell morphology under light microscope. However, the PHY106-CHBV group showed typical apoptotic features with radial cell shape, cell shrinkage, aggregation and sparse cell distribution (A3). To further verify this, we stained cells with Hoechst 333258 and observed cell nuclear morphology under a fluorescence microscope (Figure 3(B)). Similarly, in the two control groups, the nuclei showed normal features of Hoechst 333258 staining with homogenous staining and clear borders (B1 and B2). In the PHY106-CHBV group, on the contrary, the nuclei showed apparent apoptotic features of irregular nuclear border, nuclear shrinkage or fragmentation, chromatin condensation and apoptotic bodies (B3).

### Analysis of PHY106-CHBV expression induced apoptosis by flow cytometry

**Figure 4.**
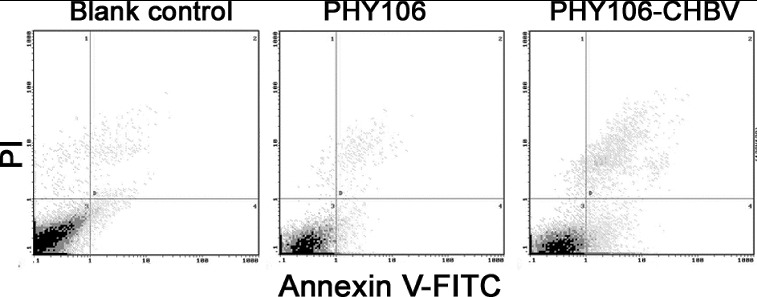
Flow cytometry analysis of cell apoptosis in NHMC cells 48 h after transfection. FITC Annexin V/PI staining. Representative analyses from three experiments.

Annexin V/PI combined with flow cytometry is another effective and sensitive way to analyse cell apoptosis. PHY106-CHBV expression-induced apoptosis was further verified by flow cytometry. Staining with FITC Annexin V in conjunction with PI allows us to distinguish early apoptosis, late apoptosis and dead cells: FITC Annexin V and PI negative (viable or no measurable apoptosis); FITC Annexin V positive and PI negative (early apoptosis, membrane integrity is present) and FITC Annexin V and PI positive (end stage apoptosis and death). The representative results of apoptosis are shown in Figure 4. Cells in the fourth quadrant (lower right) were early apoptosis cells. The early apoptosis rate in the PHY106-CHBV group (5.84% ± 1.70%) was significantly higher than the blank control group (1.09% ± 0.55%) and the PHY106 group (1.58% ± 0.67%) (*P* < 0.01).

Taken together, these results demonstrate that PHY106-CHBV expression induces NHMC cell apoptosis and that apoptosis increase is an important factor impairing cell viability and proliferation.

### Effects of PHY106-CHBV expression on Bax and Bcl-2 mRNA expression in NHMC cells

**Figure 5.**
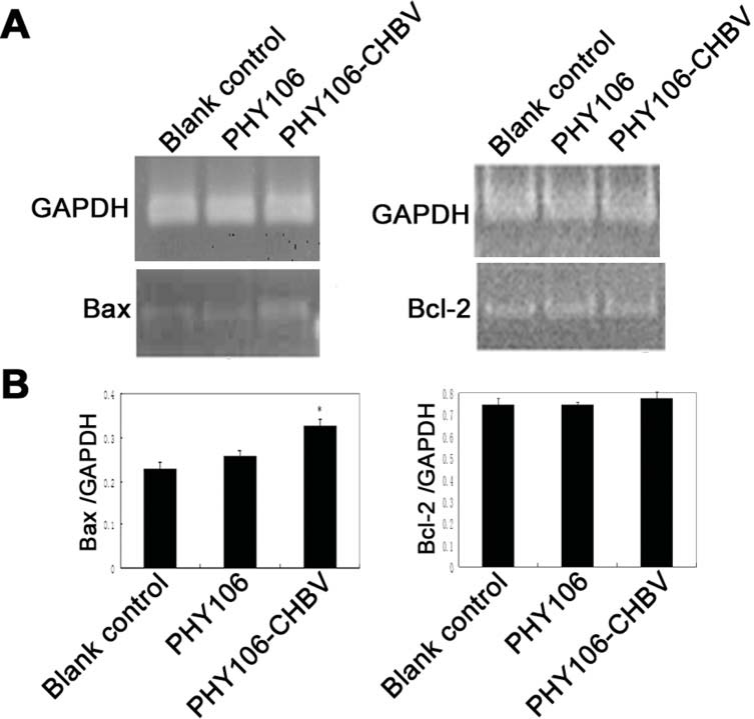
RT-PCR analysis of the expression levels of Bax and Bcl-2 mRNA 48 h after PHY106-CHBV transfection. (A) The PCR products of Bax and Bcl-2 were analysed on 1.5% agarose gel; GAPDH was used as an internal control; the grey value of each lane represents the mRNA expression level of Bax and Bcl-2. (B) Quantitative comparison of the grey value among the blank control group, the PHY106 group and the PHY106-CHBV group. *P < 0.05.

Bax and Bcl-2 are members of the Bcl-2 family of proteins that regulate apoptosis. Bax is a pro-apoptotic protein, while Bcl-2 is an anti-apoptotic protein. In order to see whether the Bcl-2-mediated apoptotic pathway was activated in PHY106-CHBV-induced apoptosis, we measured the expression levels of Bax and Bcl-2 mRNA at 48 h after transfection. The RT-PCR result is shown in Figure 5. After normalization to GAPDH, the grey values of Bax of the blank control group, PHY106 group and PHY106-CHBV group were 0.22 ± 0.02, 0.25 ± 0.02 and 0.32 ± 0.02, respectively. The expression level of Bax in the PHY106-CHBV group was significantly higher than in the control groups (*P* < 0.01). As for Bcl-2, there was no significant difference among the blank control group (0.75 ± 0.03), PHY106 group (0.75 ± 0.01) and PHY106-CHBV group (0.77 ± 0.03) (*P* > 0.05).

**Figure 6.**
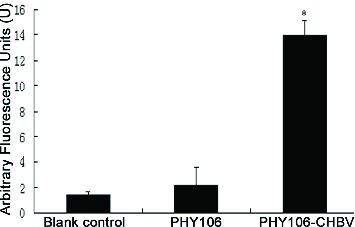
Effect of PHY106-CHBV expression on the activity of Caspase-3 in NHMC cells 48 h after transfection. The data are presented as 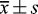 of three independent experiments. *P < 0.05 (SPSS 17.0).

### PHY106-CHBV expression activates Caspase-3 in NHMC cells

Caspase-3 is an effector caspase downstream of the apoptotic pathway and plays an important role in carrying out the cell death programme. To further verify the activation of the apoptosis pathway, Caspase-3 activity was also detected at 48 h after transfection (Figure 6). The arbitrary fluorescence units of the blank control group, the PHY106 group and the PHY106-CHBV group were 0.92 U ± 0.62 U, 1.75 U ± 1.3 U and 10.84 U ± 6.31 U, respectively. Statistically, the Caspase-3 activity in the PHY106-CHBV group was significantly higher than that in the control groups (*P* < 0.01). Collectively, our data suggest that PHY106-CHBV induces apoptosis by triggering a pathway of Bax up-regulation and Caspase-3 activation.

### Comparative analysis

Renal mesangial cells play a critical role in maintaining the structure and function of glomerular tufts and in regulating glomerular filtration by their contractility. In diabetic nephropathy, high glucose induces the apoptosis of renal tubular epithelial cells, glomerular mesangial cells and endothelial cells. In them, the apoptosis of glomerular mesangial cells is the most important and is closely correlated with the progression to diabetic glomerulosclerosis.[6,7] Whether HBV can directly cause apoptosis of mesangial cells has not been reported in the literature. In the present study, we found that PHY106-CHBV could be expressed in NHMC with high levels of HBsAg and HBeAg expression. This ectopic expression led to cell proliferation inhibition and cell apoptotic loss in NHMC cells (Figures 3 and 4).

Previous studies have shown that the pathogenesis of a variety of renal diseases is closely correlated with cell apoptosis and changes of apoptotic genes.[8,9] Sun et al. [10] detected the expression of Bcl-2 and Bax in the histopathological analysis of HBV-GN patients with immunohistochemistry. Apoptosis, under the control of genetic programs, is a multi-gene regulated and tightly orchestrated event. The Bcl-2 family is one of the closely related gene families in the regulation of cell apoptosis. It plays a fundamental role in the mitochondria-involved apoptosis pathways, mainly through the regulation of the intrinsic pathway by controlling mitochondrial membrane permeability and the release of the pro-apoptotic factor, cytochrome C. Bcl-2 family members share 1–4 homology domains termed the Bcl-2 homology (BH) domain (named BH1, BH2, BH3 and BH4). The BH3 domain is a pro-apoptotic domain, while the BH4 domain is the unique structure in anti-apoptotic proteins. Bcl-2 proteins are grouped into two classes. One class includes the ones that inhibit apoptosis (Bcl-2, Bcl-xl, Bcl-w, Mcl-1, etc.). Among them, the Bcl-2 gene, located in the short arm of chromosome 18, is currently recognized as the most important anti-apoptotic gene. It plays an important role through the mechanism of oxidative damage. The other class is the Bcl-2 proteins that promote apoptosis (Bax, Bak, Bad, Bid, Bim, etc.). These pro-apoptotic proteins all contain the BH3 domain. The Bcl-2-associated X protein, or Bax, including Bax-α, Bax-β and Bax-γ, promotes apoptosis by competing with Bcl-2. The Bcl-2 proteins can form hetero- or homo-dimers. When the expression of Bcl-2 anti-apoptotic protein is elevated in cells, the Bcl-2/Bax hetero-dimer is formed and cell apoptosis is inhibited. On the other hand, when the expression of Bax pro-apoptotic protein is elevated in cells, the Bax/Bax homo-dimer is formed and cell apoptosis is promoted.[11–14]

There are mainly two apoptotic pathways: the extracellular pathway, in which the extracellular signals activate caspases, and the intracellular (or the mitochondria) pathway, in which intracellular signals such as caspase activators released by mitochondria activate caspases. Caspases are cysteine-dependent aspartate-specific proteases and play central roles in the transduction of apoptotic signals. There are two types of Caspases: initiator Caspases, including Caspase 8, 10, 9, and effector Caspases, including Caspase 3, 6, 7. Effector Caspases are activated by initiator Caspases through proteolytic cleavage. These active effector Caspases then proteolytically degrade a host of intracellular proteins to carry out the apoptosis programme.[15,16] In the mitochondria pathway, the ratio of protein Bax to protein Bcl-2 regulates the permeability of the mitochondrial membrane and the release of cytochrome C. Once cytochrome C is released, it transduces the apoptotic signal to Caspase-9 which in turn activates the effector Caspases and initiates the cascade reaction of Caspases.[17,18] In our study, RT-PCR results showed that the expression of the pro-apoptotic Bax gene was up-regulated by PHY106-CHBV expression. Furthermore, the effector Caspase-3 was also activated. These data demonstrate that PHY106-CHBV expression up-regulated pro-apoptotic Bax gene expression and activated the effector Caspase-3, leading the NHMC cells to undergo apoptosis.

Apoptosis plays an important role in maintaining the homeostasis of the body. Under normal physiological conditions the number and morphology of mesangial cells remain relatively stable. However, when challenged with injury factors, mesangial cells undergo excessive apoptosis, causing abnormal proliferation of mesangial cells and excessive extracellular matrix generation and accumulation, resulting in glomerular mesangial matrix proliferation, and ultimately leading to the occurrence of glomerular sclerosis. Our findings confirm that HBV can induce apoptosis, and provide experimental evidence that apoptotic cell loss might be a crucial mechanism in HBV-GN pathogenesis and further provide new therapeutic targets for prevention and treatment of HBV-GN.

## Conclusions

The aim of this study was to investigate the effect of recombinant full-length C genotype HBV on NHMC *in vitro*. After PHY106-CHBV transfection, levels of HBsAg and HBeAg were increased and typical apoptotic morphology was observed. The proliferation of NHMC cells was significantly inhibited after PHY106-CHBV transfection. Additionally, the percentage of apoptotic cells, the expression level of Bax mRNA and the activity of Caspase-3 were significantly higher in the NHMC cells transfected with PHY106-CHBV. Our data suggest that HBV may induce apoptosis of NHMC cells through increasing the expression of the pro-apoptotic protein Bax and activating Caspase-3.
